# Hepatic Parenchymal Preservation Surgery: Decreasing Morbidity and Mortality Rates in 4,152 Resections for Malignancy

**DOI:** 10.1016/j.jamcollsurg.2014.12.026

**Published:** 2014-12-27

**Authors:** T Peter Kingham, Camilo Correa-Gallego, Michael I D'Angelica, Mithat Gönen, Ronald P DeMatteo, Yuman Fong, Peter J Allen, Leslie H Blumgart, William R Jarnagin

**Affiliations:** 1 Hepatopancreatobiliary Surgery, Memorial Sloan Kettering, New York, NY; 2 Epidemiology and Biostatistics, Memorial Sloan Kettering, New York, NY

**Keywords:** colorectal cancer, liver metastases, hepatectomy

## Abstract

**Background:**

Liver resection is used to treat primary and secondary malignancies. Historically, these procedures were associated with significant complications, which may affect cancer-specific outcome. This study analyzes the changes in morbidity and mortality after hepatic resection over time.

**Study Design:**

Records of all patients undergoing liver resection for a malignant diagnosis from 1993 to 2012 at Memorial Sloan Kettering were analyzed. Patients were divided into early (1993-1999), middle (2000-2006), and recent (2007-2012) eras. Major hepatectomy was defined as resection of 3 or more segments. Univariate and multivariate analyses were made with t-tests or Mann-Whitney tests.

**Results:**

3,875 patients underwent 4,152 resections for malignancy. The most common diagnosis was metastatic colorectal cancer (n=2,476, 64% of patients). Over the study period, 90-day mortality rate decreased from 5% to 1.6% (p<0.001). Perioperative morbidity decreased from 53% to 20% (p<0.001). The percentage of major hepatectomies decreased from 66% to 36% (p<0.001). The rate of perioperative transfusion decreased from 51% to 21% (p<0.001). The spectrum of perioperative morbidity changed markedly over time, with abdominal infections (43% of complications) overtaking cardiopulmonary complications (22% of complications). Peak postoperative bilirubin (OR 1.1, p<0.001), blood loss (OR 1.5, p=0.001), major hepatectomy (OR 1.3, p=0.031), and concurrent partial colectomy (OR 2.4, p<0.001) were independent predictors of perioperative morbidity. The mortality associated with trisectionectomy (6%) and right hepatectomy (3%) remained unchanged over time.

**Conclusions:**

Morbidity and mortality rates after partial hepatectomy for cancer have decreased substantially as the major hepatectomy rate dropped. Encouraging parenchymal preservation and preventing abdominal infections are vital for continued improvement of liver resection outcomes.

## Introduction

Liver resection is the most effective treatment for several malignant diseases and employed worldwide today. Historically, a major limitation of liver surgery has been the high morbidity and mortality related to blood loss and loss of functional liver. Recently, sharp reductions in perioperative mortality have been reported, from 10% in the 1980s to below 4%.(1-3) These improvements have resulted from better patient selection and perioperative management. The focus on intraoperative parenchymal preservation has led to substantial reductions in estimated blood loss (EBL), blood product transfusion, and postoperative liver failure.(4, 5) This transformation in the safety profile of hepatic resection is largely responsible for its emergence as an effective cancer therapy.

The mortality rate for major liver resections, however, such as right trisectionectomy, remains as high as 9%.(3, 6) Furthermore, there are serious complications associated with liver surgery, many of which are procedure-specific, but external factors play an increasingly important role. Co-morbid medical conditions as well as the current trend of extensive preoperative systemic chemotherapy with its impact on perioperative outcome(7) are important examples. Continued efforts to improve perioperative morbidity rates are warranted. Also, there is evidence that perioperative morbidity is a powerful predictor and possible cause of adverse disease-specific survival.(8, 9) In that light, reducing perioperative morbidity assumes even greater importance.

The type of morbidity seen after liver resection varies considerably, ranging from liver-specific, such as liver dysfunction or bile leak, to cardiopulmonary, gastrointestinal, renal, or infectious. Correcting this wide spectrum of complications is a challenge because multiple specific areas must be targeted. The present report is an analysis of 4,152 hepatic resections for malignancy over 19 years at a single center, focusing on trends in perioperative outcome variables and changes in practice.

## Methods

The liver resection database at Memorial Sloan Kettering (MSK) was queried to identify patients undergoing hepatectomy for malignancy from 1993 to 2012. The MSK Institutional Review Board provided a waiver from IRB review and HIPAA Authorization. Patients were divided into 3 groups of similar size according to time period [early (1993-1999), middle (2000-2006), and recent (2007-2012)].

The general approach to patients under consideration for liver resection has been previously documented.(10) CT, MR, or PET imaging were used for preoperative radiologic evaluations, and intraoperative ultrasound was used in all cases. Portal inflow occlusion (Pringle maneuver) was used frequently, in 5- to 15-minute intervals. Control of portal, arterial, and biliary in-flow pedicles was performed extrahepatically or intrahepatically, depending on the resection planned, disease location, and surgeon preference. Low central venous pressure (<5 mmHg) was used in all cases when feasible. Parenchymal division was performed via a clamp-crushing technique with sutures or clips to control intrahepatic biliary and vascular structures; more recently, thermal bipolar devices were added. Postoperatively, patients were managed for 12-24 hours in the recovery room and then transferred to the surgical ward, unless clinical factors dictated a monitored setting. Red blood cell transfusions were guided by patient hemodynamic status in combination with Hgb level (<8 mg/dL).

Liver resections were quantified by number of segments resected using the Brisbane 2000 terminology of hepatic anatomy and resection:(11) enucleations (0), wedge resection and formal segmentectomy (1), sectionectomy (left lateral, right anterior or posterior, 2), left hepatectomy (3), right hepatectomy (4), and extended hepatectomy (5). Major hepatectomy was defined as resection of ≥three segments. For our data, “perioperative” referred to the time period that included both operation and recovery room. Complications from 1993 to 2002 were identified retrospectively. From 2002 to 2012 complications were entered prospectively. All complications were graded using a score of 1 to 5.(12)

Complications were analyzed per patient and per resection. Major complications were defined as ≥grade 3. Liver dysfunction was defined by presence of at least one from the following: postoperative prolonged hyperbilirubinemia without obstruction or leak, prolonged coagulopathy, ascites (drainage >500 mL/day), or encephalopathy with hyperbilirubinemia.(5) Steatosis was defined as percentage of hepatocytes containing lipid vesicles divided by total number of hepatocytes. It was graded using the Kleiner-Brunt histologic scoring system, (13) where moderate steatosis was defined as 5 - 33%. Mortality was determined at 90-days post procedure.

Summary statistics are reported as median and interquartile range (IQR) for continuous variables, unless otherwise specified. Categorical variables are reported as percentages. Comparisons are made with t-tests or Mann-Whitney tests depending on type of distribution for continuous variables. Categorical variables are compared with Chi-squared or Fisher's exact test depending on number of observations. Multivariate analyses used logistic regression. All reported p values were two-tailed and those ≤0.05 were considered significant. All analyses were conducted using Stata/IC 12.1 (StataCorp LP, College Station, TX).

## Results

### Patient demographics and pathology

Four thousand two hundred two patients underwent 4,480 hepatic resections for benign and malignant conditions from 1993 to 2012. Of these, 3,875 (92.2%) patients underwent 4,152 (92.7%) resections for malignancy (Table 1). The most common comorbidities were hypertension (13%), ischemic heart disease (7%), and diabetes mellitus (5%). Two hundred five patients underwent preoperative portal vein embolization (PVE; 11% of patients with available data describing PVE status). The largest tumor per patient was 3.7 cm (IQR 2.2-6.5).

The median operating room time was 232 minutes (IQR 173-300). Portal inflow occlusion (Pringle maneuver) was used in 77% of patients with median time of 31 minutes (IQR 21-45). Of the 2,494 cases with available perioperative transfusion records, 10% received blood, 8% received fresh frozen plasma, and 2% received platelets. Liver resection was combined with resections of other organs in 976 (25.2%) patients. This included colon (9%), portal lymph nodes (7%), and extrahepatic bile duct (3%). One thousand thirty-nine (39.8%) of 2,610 patients had steatosis; 27% of these patients had moderate-to- marked steatosis. The median length of stay after liver resection was 7 days (range 6-10). Thirty-day mortality was 1%, 60-day mortality was 2%, and 90-day mortality was 3%.

### Trends over time

The percentage of major hepatectomies decreased from 65.6% to 35.8%, p<0.001, over the three eras (Table 2). The largest decrease was right trisectionectomies, from 27.8% to 6.7%. The median size of resected tumors decreased from 4.5 cm to 3.5 cm to 2.5 cm in each era (p=0.01). The EBL and complication percentages both significantly decreased over time. The percentage of resections combined with ablation increased from 0.6% (n=8) in the first era, to 4.4% (n=65) in the second era, to 19.2% (n=271) in the recent era, p<0.001. In addition, the percentage of minimally invasive cases increased, from zero (0%) in the first era to 70 (5%) in the third era, p<0.001. Ninety-two percent of the resections were R0 (of 2,840 resections with specific margin reported). Of note, the percentage of tumor-involved margins did not change, as less liver was resected using parenchymal-preserving techniques − 9% in the early and recent eras. One hundred twenty-two patients (3% of patients with available data on reoperation) had a second operation to treat a complication within 30 days of the original procedure. This percentage remained constant over time (2-3%). The overall transfusion percentage of blood products over the duration of the hospital stay decreased from 51% in the early era, to 21% in the recent era (p<0.001). Neoadjuvant chemotherapy use increased from 13% to 36% to 55% over three eras (p<0.001).

### Complications

**Figure 1:**
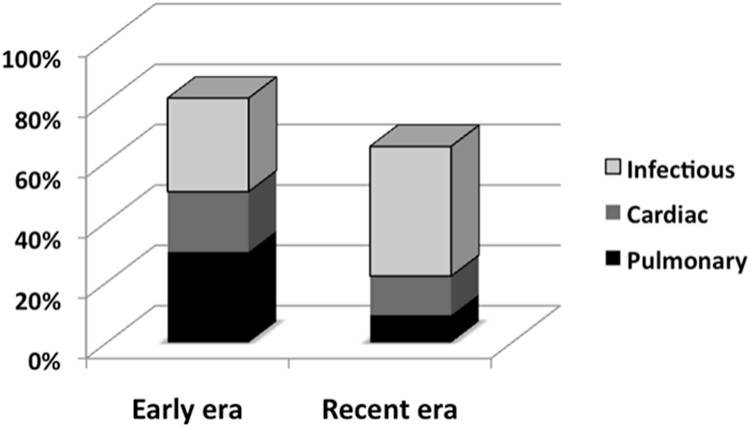
Cause of complication in patients in the early and recent era who had a reported complication (p<0.001).

At least one complication occurred in 1,462 resections (35%, Table 3). The most common complication was abdominal infection (wound infections, abdominal abscesses, and bilomas), occurring in 11.6% of all resections, but with a significant decrease over time, from 16.5% to 8.6% (Table 4, p<0.001). The pattern of perioperative morbidity significantly changed over the study period (Figure 1). In the first era, 50% of complications that occurred were pulmonary (30%) or cardiovascular (20%) in nature. This decreased to 22% (9% and 13%, respectively) for the recent period. By contrast, abdominal infections rose from 31% to 43% of all complications in the recent period. Of note, however, the morbidity rate differed by diagnosis. Over the entire study period, major and minor complication rates were similarly high for hilar cholangiocarcinoma (49%) and metastatic colorectal cancer (42%), but were lower for hepatocellular cancer (28%), gallbladder cancer (27%), and intrahepatic cholangiocarcinoma (16%, p<0.001).

Factors associated with complications were analyzed in univariate and multivariate analyses. Peak postoperative bilirubin (OR 1.1, p<0.001), EBL (OR 1.5, p=0.001), perioperative transfusion (OR 1.5, p<0.001), major hepatectomy (OR 1.3, p=0.031), and hepatectomy combined with a colorectal resection (OR 2.4, p<0.001) were independent predictors of postoperative morbidity on multivariate analysis. Minimally invasive surgery, use of a drain, a positive margin, and Pringle time greater than 25 minutes all lost significance on multivariate analysis. Since postoperative bilirubin level may be influenced by multiple factors unrelated to liver dysfunction, the analysis was repeated excluding this variable. In this model, perioperative transfusion (OR 2.0), blood loss (OR 1.6), major hepatectomy (OR 1.7), and hepatectomy combined with a colon resection (OR 1.6) remained independent predictors of postoperative complications. Data from synchronous colorectal resections showed almost twice the risk of abdominal infectious complications (21%) when compared to staged procedures (11%, p<0.001). In addition, preoperative jaundice was significantly associated with infectious complications, with an odds ratio of 2.1 with preoperative bilirubin greater than 3 (95% CI 1.27-3.6; p=0.004). Postoperative liver dysfunction decreased in all cases from 3% in the first era to 1% in the recent era (p<0.001). For major resections, postoperative liver dysfunction rates decreased from 5% to 3% (p<0.001). On multivariate analysis, transfusion showed a strong association with the development of liver dysfunction, followed by number of segments resected, number of medical comorbidities, and preoperative abnormalities in bilirubin and albumin.

In parallel with fewer major hepatectomies performed over time, the 90-day mortality rate for all cases decreased from 5.2% in the early era to 1.6% in the recent era, p<0.001. The median time to postoperative death was 36 days (IQR 18-64). The proximate cause of postoperative mortality was unknown in 31 patients; in the remaining patients, liver failure (n=26), multi-system organ failure (n=18), intra-abdominal abscess (n=4), and hemorrhage (n=7) were clearly identified as causative factors. Forty-one percent of patients with an unknown cause of mortality had ≥4 segments resected in the first era and had documented evidence of liver failure. Although these patients did not have a clearly documented cause of death, it is likely that most were liver-related. The majority of perioperative deaths (79%) occurred after major resections. Only 2 of the 31 (6.5%) mortalities after resection of <3 segments were due to liver failure, which was 0.1% of 2,027 patients subjected to a minor resection. In comparison, 23 (24.2%) of the 95 patients that died after a major resection had liver failure (p=0.06), representing 1.1% of all major hepatectomies (n=2125).

The overall mortality rate for major resections was 4.5% (96/2125). Although the overall mortality rate clearly decreased over the three eras, it remained high and unchanged for right and left trisectionectomies (6%) and right hepatectomies (3%) within the recent era (Table 5). As the percentage of major hepatectomies decreased over time, the 90-day mortality rate for all resections decreased from 5.2% in the first era to 1.6% in the recent era (p<0.001; Table 2). Peak postoperative bilirubin ≥7 mg/dL was associated with a mortality rate of 17% (OR 10; 95% CI 6.24-16.1, p<0.001). The mortality rate for peak postoperative bilirubin ≥7 mg/dL in postoperative days 1 to 3 was 14% (n=10), days 4 to 7 was 17% (n=7), and later than day 7 was 35% (n=6, p=0.1). The sensitivity of a bilirubin level ≥7 mg/dL for predicting mortality was 38%, with specificity of 97%.

## Discussion

Liver surgery for the treatment of primary and secondary malignancy has evolved dramatically over the last twenty years. Notable changes include increased utilization of parenchymal-sparing resections and low intraoperative central venous pressure, better patient selection, use of 2-stage resections in patients with advanced disease, utilization of liver volumetric assessment of future liver remnant, improvements in perioperative management, and increased utilization of preoperative chemotherapy. In concert with these changes, the mortality associated with hepatic resection has significantly decreased.(14, 15) Two large studies reported that despite the higher proportion of older patients with an attendant increase in comorbidities and abnormal livers, both complication and mortality rates have decreased with the recent era.(3, 4)

The present study showed a significant decrease in overall complications from 53.2% to 19.9% over the past two decades. Furthermore, the 90-day mortality rate in this series decreased from 5.2% to 1.6%. Much of the decrease in mortality was likely related to the decreasing number of major resections. Evidence in support of this is the persistently high mortality rates over the three eras for resections associated with substantial parenchymal sacrifice (right and left liver trisectionectomy, and right hepatectomy). Thus, continued efforts to pursue parenchymal-sparing resections will likely translate over time into further reduction in operative mortality.

Despite declining mortality rates, the morbidity associated with liver resection, although also declining, remains substantial. It is important to assess the pattern of complications because the oncologic sequelae of perioperative morbidity can be significant. In our series, abdominal infections had become the most common complication by the recent era. We previously analyzed the oncologic outcomes of 1,067 patients with liver resections at MSK to treat metastatic colorectal cancer.(16) A subgroup analysis of patients with low clinical risk score revealed that complications were associated with a significant reduction in disease-specific survival and disease-free survival. Additionally, in patients with metastatic colorectal cancer enrolled in a randomized trial of intraoperative hemodilution, postoperative complications were associated with significant decreases in recurrence-free survival (69 to 23 months, p<0.001) and overall survival (74 to 28 months, p<0.001).(9) The mechanism that translates perioperative morbidity into reduced cancer-specific survival is unknown, although results from several animal models have suggested that infectious complications increase neutrophil numbers and activation, and may in turn promote liver metastasis.(17, 18)

Given the independent association of the number of segments resected with complications and mortality, hepatic preservation appears to have played a major role in reducing complications. Due to changes in patient selection criteria for resection and adjuvant/neoadjuvant therapies over the last 20 years, it is difficult to determine the contribution that parenchymal-preserving resection has played in long-term outcomes, particularly for the subgroup with colorectal cancer. However, the strong association of complications with adverse survival, and the strong association of hepatic preservation with a decrease in complications make a compelling argument. We found a decrease in major hepatectomies from 65.6% to 35.8% over 19 years (p<0.001), with a corresponding decrease in the median number of segments resected from 4 to 2 (p<0.001). This change in approach to resection appears to be largely responsible for the observed decrease in overall mortality, given that the mortality rate associated with extended liver resections remained constant over the entire study period. In support of this conclusion, a recent series reported a 90-day mortality rate of 9% for major hepatectomies in patients that required PVE.(6) Given the persistently high mortality rate associated with major hepatectomies, a parenchymal-preserving approach, when possible, is important for limiting morbidity and mortality, and in so doing, may improve cancer-related outcomes. Furthermore, data from the present study show that parenchymal-sparing resections do not compromise oncologic integrity.

One of the sequelae of removing less liver is that the transfusion rate decreased from 51% to 21% (p<0.001) and liver dysfunction for all cases decreased. One factor that likely contributed to the increasing use of parenchymal preservation was the expansion of ablation in patients with colorectal metastases. The use of ablation combined with resection has allowed preservation of parenchyma in the future liver remnant, thereby expanding the indications for operative therapy in patients with advanced disease. Ablations were involved in 19.2% of cases in the recent era (increased from 0.6% of the early era). This strategy appears to be oncologically reasonable with small tumors, as the local recurrence rate for tumors <1 cm is 6%, with over 90% of recurrences occurring elsewhere in the liver or systemically. The lower EBL as fewer segments were resected is important, as EBL and transfusion are both associated with perioperative complications. Other factors in this regard included postoperative hyperbilirubinemia, concomitant colon resection, and ≥3 segments resected. Recently, Aloia et al, using the National Surgical Quality Improvement Program (NSQIP) dataset, identified similar factors in 2,313 hepatectomies.(19) Given the limitations inherent to NSQIP, only the 30-day mortality rate could be analyzed. It was 2.5%, along with a 30-day major morbidity rate of 19.6%. In our series the mortality rate tripled from 30 to 90 days.

In addition to hepatic preservation, other preoperative and intraoperative strategies can affect outcomes after liver surgery. Portal vein embolization is a valuable tool to optimize size of future remnant in patients for major liver resection and is now practiced widely (7% of patients in the recent time period). Patients with underlying liver disease, including fibrosis, cirrhosis, and chemotherapy-associated liver injury, appear to benefit most, as demonstrated in a recent randomized trial (20). Associating liver partition and portal vein ligation for staged hepatectomy (ALPPS) has been suggested as an alternative technique to decrease liver failure, but it is likely applicable in only a few percent of cases and is associated with a prohibitively high mortality rate (>10% in-hospital mortality rate).(6) An effective PVE is usually sufficient and has lower morbidity and mortality.(6, 21) Intraoperatively, maintenance of a low central venous pressure assists with minimizing blood loss, and is thus an important consideration for a liver surgery anesthesia team.

Liver failure is the most serious liver-specific complication posthepatectomy. Liver failure has been identified in as many as 10% of patients in some series.(22) One study reported posthepatectomy total bilirubin level greater than 7 mg/dL had a sensitivity of 93% and specificity of 94% for predicting liver-related death.(23) We found a sensitivity of 38% but specificity of 97% for predicting overall survival with this postoperative bilirubin level. We found that the peak postoperative bilirubin greater than 7 mg/dL had an odds ratio of 10 for postoperative mortality with a 17% mortality rate. The immediate postoperative platelet count has also been associated with delayed normalization of liver function. In the present study, only one of 70 patients with postoperative platelet count <100,000 died, so this did not apply.

The limitations of this study are several. First and foremost, much of the data is retrospective and there are missing values for some patients, especially in the earliest era. Complications have been recorded prospectively only since 2002. The value of this large database, however, is that it demonstrates a shift in technique and pattern of complications over the last 19 years, with a focus on parenchymal preservation. This approach to liver surgery should be reinforced as a method of decreasing morbidity and mortality, which in turn may lead to improvements in cancer-specific outcome. Despite the increased safety of these procedures, however, complications are still common. Abdominal infections are the most common complication of modern liver surgery, and should be targeted to further improve liver surgery outcomes.

## Figures and Tables

**Table 1:** Demographics and Comorbidities Associated with 4,152 Hepatic Rresections

Patient data	Number
Age, y (range)	61 (51-70)
Sex, % male	53%
BMI, kg/m^2^ (IQR)	26 (23-30)
Comorbidity, n (%)	
1	620 (14.9%)
2	278 (6.7%)
≥3	113 (2.7%)
Smoking history	2671 (64.3%)
Chronic hepatitis B or C	186 (4.5%)
ASA score (n=2,270)	
1	17 (0.7%)
2	1056 (46.5%)
3	1197 (52.7%)
Preoperative, n (%)	
Bilirubin, mg/dL (n=4,043)	
0-1	3579 (88.5%)
1.1-2	348 (8.6%)
2.1-3	44 (1.1%)
3.1-10	43 (1.1%)
>10	29 (0.7%)
Albumin, g/dL	
<4	1020 (24.6%)
<3.5	249 (6.0%)
Platelets	
<150,000/uL	539 (13.0%)
<100,000/uL	76 (1.8%)
Hgb	13 (12-14)
Neoadjuvant chemotherapy	1414 (34.0%)
Metastatic colorectal cancer	2476 (59.6%)
Primary liver	623 (15.0%)
Extrahepatic biliary	334 (8.0%)

IQR, interquartile range; ASA, American Society of Anesthesiologists; Hgb, hemoglobin.

**Table 2:** Surgical Procedures Grouped by Era

	1993-1999 n=1275	2000-2006 n=1465	2007-2012 n=1412	p Value
Major hepatectomy, ≥3 segments	65.6% (836)	53.5% (784)	35.8% (505)	<0.001
Segments resected, median (IQR)	4 (2-5)	3 (2 - 4)	2 (1-4)	<0.001
Left hepatectomy	6.9% (88)	8.6% (126)	8.4% (119)	
Left trisectionectomy	6.1% (78)	3.0% (44)	2.5% (36)	
Right hepatectomy	19.9% (254)	19.1% (280)	13.6% (192)	
Posterior sectionectomy	4.8% (61)	5.9% (87)	5.0% (71)	
Right trisectionectomy	27.8% (355)	16.6% (244)	6.7% (95)	
Resection combined with ablation	0.6% (8)	4.4% (65)	19.2% (271)	<0.001
Estimated blood loss, median (IQR)	650 (310 - 1110)	400 (200 - 750)	300 (200 - 565)	0.003
Complications	53.2% (679)	34.3% (502)	19.9% (281)	<0.001
Major complications	13.2% (169)	11.2% (164)	9.8% (138)	0.017
Readmission, 30 d	13.2% (169)	12.3% (180)	13.6% (192)	0.6
ASA score (range)	-	2 (2-3)	3 (2-3)	<0.001
90-d mortality	5.2% (66)	2.3% (34)	1.6% (22)	<0.001

For major complications, grading of complications was available for 931, 1,301, and 1,412 patients in each era, respectively. IQR, interquartile range; ASA, American Society of Anesthesiologists.

**Table 3:** Postoperative Complications

Organ system	Specific complication	n	Percentage (of total resections)
Liver/biliary		236	5.7%
	Hepatic insufficiency/failure	82	2.0%
	Sterile perihepatic fluid collections	161	3.9%
	Biliary stricture	6	<1%
	Cholangitis	3	<1%
Pulmonary		311	7.5%
	Pleural effusion	188	4.5%
	Atelectasis	57	1.4%
	Respiratory insufficiency/failure	58	1.4%
	Pulmonary embolism	40	1.0%
Cardiovascular		239	5.8%
	Arrhythmia	129	3.1%
	DVT	46	1.1%
	CHF	24	<1%
	Angina pectoris/MI	16	<1%
	Cardiac arrest	12	<1%
	Stroke/TIA/CNS hemorrhage	11	<1%
	Pericarditis/pericardial effusion	2	<1%
Genitourinary		99	2.4%
	Renal insufficiency/failure	56	1.3%
	Urinary retention	48	1.2%
Gastrointestinal		190	4.6%
	Ileus	121	2.9%
	Bowel obstruction	36	<1%
	GI hemorrhage	21	<1%
	Colitis (includes *C. diff*)	28	<1%
	Bowel perforation	8	<1%
Infection		585	14.1%
Abdominal		483	11.6%
	Wound infection	255	6.1%
	Perihepatic abscess	214	5.2%
	Bile leak/biloma	105	2.5%
Non-abdominal		203	4.9%
	Sepsis/bacteremia	61	1.5%
	UTI	99	2.4%
	Pneumonia	79	1.9%
Miscellaneous		286	6.9%
	Perioperative hemorrhage	38	<1%
	Delirium	26	<1%
	Wound breakdown/fascial dehiscence	33	<1%
	Ascites	44	1.0%
	Pump dysfunction	27	<1%
	Multisystem organ failure	18	<1%
	Other	135	3.3%

Resections with multiple associated complications in the same category were counted only once. DVT, deep-vein thrombosis; CHF, congestive heart failure; TIA, transient ischemic attack; CNS, central nervous system; GI, gastrointestinal; UTI, urinary tract infection.

**Table 4:** Summary of Complications after All Resections over 3 Eras, and of Complications after Major Resections over 3 Eras

	Era						
	1993 - 1999		2000 - 2006		2007 - 2012		p Value
	n	%	n	%	n	%	
All resections							
Total cases	1,275		1,465		1,412		
Total cases with complications	679	53.2%	502	34.3%	281	19.9%	p < 0.001
Abdominal infections	211	16.5%	151	10.3%	121	8.6%	p < 0.001
GI	98	7.7%	52	3.5%	40	2.8%	p < 0.001
Non-abdominal infections	120	9.4%	64	4.4%	19	1.3%	p < 0.001
Liver/Biliary	109	8.5%	97	6.6%	30	2.1%	p < 0.001
Pulmonary	208	16.3%	79	5.4%	24	1.7%	p < 0.001
Cardiovascular	133	10.4%	70	4.8%	36	2.5%	p < 0.001
GU	64	5.0%	22	1.5%	13	0.9%	p < 0.001
Major resections							
Total	836	65.6%	784	53.5%	505	35.8%	
Total with complications	491	58.7%	312	39.8%	133	26.3%	p < 0.001
Abdominal infections	168	20.1%	106	13.5%	57	11.3%	p < 0.001
Gastrointestinal	66	7.9%	29	3.7%	15	3.0%	p < 0.001
Liver/Biliary	100	12.0%	74	9.4%	22	4.4%	p < 0.001
Pulmonary	156	18.7%	58	7.4%	21	4.2%	p < 0.001
Non-abdominal infections	92	11.0%	39	5.0%	9	1.8%	p < 0.001
Genitourinary	46	5.5%	18	2.3%	8	1.6%	p < 0.001
Cardiovascular	94	11.2%	49	6.2%	16	3.2%	p < 0.001

GI, gastrointestinal; GU, genitourinary.

**Table 5:** 90-day Mortality for High-Risk Major Hepatectomies

Procedure	Era 1	Era 2	Era 3	Total
Left trisectionectomy	10.2% (8)	0% (0)	5.6% (2)	6% (10)
Right trisectionectomy	8.7% (31)	2.9% (7)	6.3% (6)	6% (44)
Right hepatectomy	4.7% (12)	3.6% (10)	2.6% (5)	4% (27)
Left hepatectomy	4.5% (4)	3.2% (4)	0.8% (1)	3% (9)
Fewer than 4 segments resected	2% (11)	2% (13)	1% (8)	1% (32)
